# Special Issue on “Advances in Image-Guided Diagnosis and Treatment of Abdominal Diseases”

**DOI:** 10.3390/diagnostics13020169

**Published:** 2023-01-04

**Authors:** Paolo Marra, Francesco Giurazza

**Affiliations:** 1 Department of Radiology, ASST Papa Giovanni XXIII Hospital, 24127 Bergamo, Italy; 2 School of Medicine and Surgery, University of Milano-Bicocca, 20126 Milan, Italy; 3 Department of Vascular and Interventional Radiology, Cardarelli Hospital, 80131 Naples, Italy

## 1. Introduction

This Special Issue is a collection of five scientific papers and five reviews concerning current topics in abdominal imaging with attention on the diagnosis and interventional management of specific diseases. Ultrasound, computed tomography, and magnetic resonance are described in different clinical situations, and their roles are emphasized in regards to the exact characterization of diseases and the interventional management that they may require. The included papers could be of interest both for general practitioners, in order to enrich their knowledge of less common topics, and for more experienced readers, to further improve their specific skills. Although imaging is the focus of the Special Issue, the content is accessible and comprehensible even for non-radiologist readers. 

Some of the articles are from experts in the field personally invited by ourselves, and all of them underwent a peer-review process with two rounds of review, with at least three experts carefully reviewing each submitted paper.

## 2. Papers Included in the Special Issue

Ultrasound imaging is commonly used as a first-line tool, but its reliability is often questioned by its non-objectivity and limited field of view. However, in many clinical situations and especially for most vascular applications, ultrasound can provide a definitive diagnosis and interesting functional data. Curti et al. [1] presented the potential role of an advanced ultrasound technique to detect type II endoleak after endovascular repair of the abdominal aorta. The patented superb microvascular imaging (SMI) technique, developed in 2014, allows the purification of the Doppler signal, removing background artifacts and noise, without affecting the vascular signal. SMI proved to be comparable to contrast-enhanced ultrasound with a higher than 90% agreement compared to the standard of reference, computed tomography angiography. Also, Collaku et al. [2] investigated the role of SMI in another interesting but very selected vascular application. The authors showed the added value of the technique, coupled with conventional color Doppler, in order to enhance the visibility of the hepatic artery in pediatric patients with liver transplants. In this clinical scenario, the reliability of ultrasound should be optimized, to avoid further unnecessary investigations with more invasive techniques such as computed tomography angiography. The main advantages of ultrasound are that it can be performed repeatedly without any concern of contrast agent administration and ionizing radiation exposure, which is remarkable in children and in the elderly.

Regarding functional assessment, ultrasound can provide information on the hemodynamic status of patients. Eke et al. [3] demonstrated that the venous waveform of liver Doppler ultrasound before cardiac surgery may predict the development of postoperative acute kidney injury. These findings may help to better select patients or to adopt pre-emptive nephroprotective strategies in patients at higher risk.

Computed tomography is the most widely used second-line technique, performed both in emergency and in routine situations. Due to its large applications in the general population, not only incidental findings constantly emerge, but also potentially useful information about subclinical conditions. Chung et al. [4] observed that unenhanced computed tomography performed for screening in an adult population can reveal the presence of hepatic steatosis related to metabolic syndrome. The degree of liver steatosis can be interpreted alongside clinical and laboratory data, to implement necessary preventive or therapeutic strategies for patients. Another relevant issue in computed tomography imaging is the optimal dose of contrast agent that should be administered to obtain the best image quality. Several protocols have been proposed, ranging from fixed or semi-fixed doses to body-weight (total or lean) adjusted ones. De Jong et al. [5] showed that a total-body-weight protocol provided more uniform attenuation values among patients, reducing the overall dose of contrast agents without a subjective sacrifice in image quality. However, it is worth mentioning that the technological development and application of artificial intelligence with deep learning in computed tomography image reconstruction contribute to a substantial reduction in the amount of administered contrast agent and radiation dose. The best protocol should be tailored to locally available technology and skills. Computed tomography angiography is the most suitable technique to provide a quick and panoramic view of vascular diseases in urgent situations. Moreover, it offers high-resolution images that can be reformatted on multiple planes in order to accurately plan interventions. Corvino et al. [6] reviewed the role of Computed Tomography Angiography in the diagnosis and endovascular treatment planning of splenic artery pseudoaneurysms.

Magnetic resonance imaging has the unique ability to characterize tissues based on their physical properties. However, for the physical principles on which it relies, it suffers confounders. The liver is probably the most studied organ of the abdomen by magnetic resonance and Pecorelli et al. [7] and Franceschi et al. [8] wrote two interesting review papers on liver signal modifications under specific circumstances. The former deals with focal liver lesion characterization in the background of iron accumulation; the latter describes the modification of liver signal intensity on T2-weighted sequences induced by a commonly used hepatocyte-specific contrast agent.

Finally, two interesting reviews presented a comprehensive overview of different imaging modalities adopted in clinical practice, respectively, for the management of portal vein thrombosis and malignant melanoma, which are two relatively uncommon but very tricky conditions. Marra et al. [9] described the wide spectrum of portal vein thrombosis appearance in pediatric patients and adults, focusing on precise disease characterization with multimodality imaging. The authors remarked on the developments in the field, especially regarding the role of invasive imaging and interventional radiology, which led to a substantial improvement in the management of this clinical entity. Ionescu et al. [10] extensively treated the possible manifestations of intra-abdominal melanoma with a brief description of imaging modalities, highlighting the superior role of positron emission tomography for whole-body staging and treatment response assessment in the era of immunotherapy.

## 3. Conclusions

In this editorial, we provided an overview of the promising articles selected for the Special Issue and hope that they will inspire further research on related topics. We would also like to express our gratitude to all the authors who submitted their valuable manuscripts, to the reviewers who provided timely and professional comments, and to the Diagnostics Editorial Office team for their support in the Special Issue’s design and curation.
